# Detection of distant relatedness in biobanks for identification of undiagnosed carriers of a Mendelian disease variant: application to Long QT Syndrome

**DOI:** 10.21203/rs.3.rs-3314860/v1

**Published:** 2023-09-15

**Authors:** Megan C. Lancaster, Hung-Hsin Chen, M. Benjamin Shoemaker, Matthew R. Fleming, James T. Baker, Grahame Evans, Hannah G. Polikowsky, David C. Samuels, Chad D. Huff, Dan M. Roden, Jennifer E. Below

**Affiliations:** 1 Division of Cardiovascular Medicine, Department of Medicine, Vanderbilt University Medical Center, Nashville, Tennessee, 37232, U.S.A.; 2 Division of Genetic Medicine, Department of Medicine, Vanderbilt University Medical Center, Nashville, Tennessee, 37232, U.S.A.; 3 Department of Molecular Physiology and Biophysics, Vanderbilt University, Nashville, Tennessee, 37232, U.S.A.; 4 Division of Cancer Prevention and Population Sciences, Department of Epidemiology, University of Texas MD Anderson Cancer Center, Houston, Texas, 77030, U.S.A.; 5 Division of Clinical Pharmacology, Department of Medicine, Vanderbilt University Medical Center, Nashville, Tennessee, 37232, U.S.A.

## Abstract

Rare genetic diseases are typically studied in referral populations, resulting in underdiagnosis and biased assessment of penetrance and phenotype. To address this, we developed a generalizable method of genotype inference based on distant relatedness and deployed this to identify undiagnosed Type 5 Long QT Syndrome (LQT5) rare variant carriers in a non-referral population. We identified 9 LQT5 families referred to a single specialty clinic, each carrying p.Asp76Asn, the most common LQT5 variant. We uncovered recent common ancestry and a single shared haplotype among probands. Application to a non-referral population of 69,819 BioVU biobank subjects identified 22 additional subjects sharing this haplotype, subsequently confirmed to carry p.Asp76Asn. Referral and non-referral carriers had prolonged QTc compared to controls, and, among carriers, QTc polygenic score additively associated with QTc prolongation. Thus, our novel analysis of shared chromosomal segments identified undiagnosed cases of genetic disease and refined the understanding of LQT5 penetrance and phenotype.

## Introduction

Most data on the impact of rare variants in Mendelian disease genes have been gathered in referral or registry populations. This approach overestimates true population impact, which is better assessed in large non-referral population cohorts, such as biobanks. Since most biobanks recruit participants regionally, there is often significant undocumented (“cryptic”) relatedness among participants. This oversampling of related individuals provides an abundance of genomic segments that are shared without recombination due to common ancestry. These identical-by-descent (IBD) segments provide an opportunity to study ungenotyped or poorly genotyped rare variants harbored within them. Because rare variants within IBD segments are shared if present in the common ancestor, IBD segments can identify likely carriers of rare variants,^1^ inform relationships and reconstruct pedigrees,^2–5^ and map causal variants, including in biobanks where relatedness is often cryptic.^6,7^

Here, we innovatively leveraged IBD segments in a large biobank to investigate a rare variant in *KCNE1* that is causal for Long QT syndrome (LQTS), a well-recognized, rare cause of syncope and sudden cardiac death (SCD) with an estimated prevalence of 1:2000.^8^ *KCNE1* mutations cause Type 5 Long QT Syndrome (LQT5 [MIM: 613695]),^9^ a subtype accounting for 1–2% of autosomal dominant congenital LQTS cases. *KCNE1* encodes a function-modifying subunit for the voltage-gated slow delayed rectifier potassium current I_Ks_,^10^ and possibly other potassium currents.^11–13^ Functional studies of the missense mutation, c.226G>A (rs74315445), resulting in p.Asp76Asn, have shown a dominant negative effect to reduce I_Ks_.^9^ However, recent registry and referral center-based studies argue that *KCNE1* variants have low penetrance (10–30%) and are not truly disease-causing, but rather function-modifying, and predispose to drug-induced forms of LQTS.^14–16^

An international consortium of 26 centers identified 89 probands with possible LQT5, 140 additional carrier relatives, and 19 cases of Jervell-Lange-Nielsen Syndrome (MIM: 612347) attributed to homozygous or compound heterozygous *KCNE1* loss of function variants.^17^ The commonest mutation was p.Asp76Asn, with 35 probands and 63 carrier relatives. Nine probands, as well as 3 carrier relatives, were identified at Vanderbilt University Medical Center (VUMC), representing a marked enrichment relative to other sites. We hypothesized that these local probands were distantly related, and that this interrelatedness would provide an opportunity to identify additional carriers in a regional biobank and to establish the impact of p.Asp76Asn.

To identify likely carriers in BioVU, we developed a novel approach, DRIVE (Distant Relatedness for Identification and Variant Evaluation) that leverages IBD to generate unbiased estimates of the role of p.Asp76Asn. First, we estimated the genome-wide relatedness among the 12 clinical p.Asp76Asn carriers and reconstructed pedigrees. We then identified the shared haplotypes spanning *KCNE1*. We used DRIVE to identify BioVU subjects who share IBD segments containing *KCNE1* with clinic carriers, and confirmed p.Asp76Asn carrier status via sequencing. Finally, we assessed electrocardiograms (ECGs) and medical records for features of LQTS. This enlarged carrier group in a hospital-based population improved power to revisit the debate about the role of *KCNE1* in LQTS, as well as to identify the interaction between p.Asp76Asn carriage and a QTc polygenic risk score (PRS) in modifying p.Asp76Asn penetrance. Our findings highlight the utility of IBD analysis in large biobanks to identify undetected carriers of rare pathogenic variants. This enables analyses of interacting effects, expansion of clinical phenotypes, and estimation of variant penetrance in a non-referral clinical population.

## Subjects and Methods

### Clinic subjects

Nine probands and three family members from the Genetic Arrhythmia Clinic at VUMC were clinically genotyped and found to be heterozygous carriers of the *KCNE1* p.Asp76Asn variant. One proband was identified incidentally in the eMERGE III (Electronic Medical Records and Genomics Phase III) sequencing study,^18^ and was subsequently referred to the Genetic Arrhythmia Clinic. All clinic carriers were of European descent.

### Biobank subjects

BioVU is the VUMC biorepository linking deidentified electronic health records (EHRs) to over 300,000 DNA samples derived from specimens about to be discarded after clinical testing.^19^ The EHR at VUMC contributes specimen-linked de-identified demographic data, clinical notes, electronic orders, laboratory measurements (including ECG data), Current Procedures Terminology (CPT) codes, and International Classification of Diseases (ICD-9 and ICD-10) codes. Currently in BioVU, 95,124 individuals have been genotyped on the Illumina Expanded Multi-Ethnic Genomic Array (MEGA^EX^), and 54,347 of these have had at least one ECG recorded.

### MEGA^EX^ Genotyping and Quality Control

Variants with >2% missingness and individuals with >3% missingness were excluded from further analysis. All BioVU subjects were projected against principal components from all populations in 1000 Genomes^20^ to determine genetic ancestries using PLINK.^21,22^ In total, 69,819 subjects clustered with the European superpopulation (EUR), 15,603 with the African superpopulation (AFR), 897 with the East Asian superpopulation (EAS), 414 with the South Asian superpopulation (SAS), and 2,466 with the Admixed Americans superpopulation, as previously described.^23^ Within groups, additional quality control was conducted using the following thresholds for inclusion: minor allele frequency (MAF) > 0.01, variant missingness < 0.05, sample missingness <0.1, heterozygosity F<0.2, Hardy-Weinberg equilibrium p-value>1×10^−10^, removal of duplicate samples, and a requirement for genetic sex to match clinical records. After these quality assessments, 69,819 EA individuals with 718,367 variants were kept in further analysis. Although *KCNE1* p.Asp76Asn was genotyped by the MEGA^EX^ array, it was excluded from downstream analysis as it did not pass MAF threshold (rs74315445 MAF=0.00011 in Europeans^24^) in quality control (QC).

The 12 clinic subjects were genotyped on the MEGA^EX^ array to generate haplotype data using the same array and following the same protocols as those in BioVU. For these 12 subjects, written informed consent for genotyping was obtained under VUMC IRB approval. Because all 12 were of European ancestry and bias can be introduced in small and highly related samples, we carried forward the set of QC-passed genetic variants in the BioVU EA dataset rather than conducting variant-based QC separately in the clinical subjects. For individual-level QC, all clinic samples’ genetic sex matched the recorded sex, heterozygosity levels were below 0.2, and all had a call rate >0.989.

### Phasing and IBD detection

Phasing, the process of determining haplotypes (the sequence of alleles on a single chromosome) from genetic data, is a critical step for accurately identifying IBD segments. SHAPEIT4^25^ was used to establish phase in genotype data from both BioVU EA subjects and clinic samples, separately. The BioVU EA dataset is sufficiently large to conduct phasing without an external reference panel. For the 12 clinic samples, the phased BioVU EA genetic data were used as the reference panel during phasing. The BioVU EA subjects and clinic samples were merged after phasing using BCFtools.^26^ We leveraged Hap-IBD,^27^ a seed-and-extension approach, to detect IBD segments efficiently on biobank-scale data. We required a minimum shared segment length of 100 contiguous genetic markers and a minimum length of 2 centimorgan (cM) as initial seeds in Hap-IBD and carried IBD segments longer than 3 cM forward in analysis to minimize analysis of erroneous segments.

### Pedigree reconstruction and distant relatedness estimation

Genome-wide IBD proportions were calculated using the method-of-moments estimation function in PLINK^21^ after removing ancestry-informative SNPs in PRIMUS.^4^ Non-directional networks of first- and second-degree related individuals were reconstructed into pedigrees using PRIMUS. In addition the length and distribution of IBD segments genome-wide were used to identify more distant relatives (up to ninth degree) by ERSA,^28^ and then passed into PADRE^2^ to generate pedigree-aware estimates of distant relatedness. Relatedness estimation and pedigree reconstruction were conducted in BioVU EA and clinic subjects separately.

### Local IBD clustering

**Figure 1.**
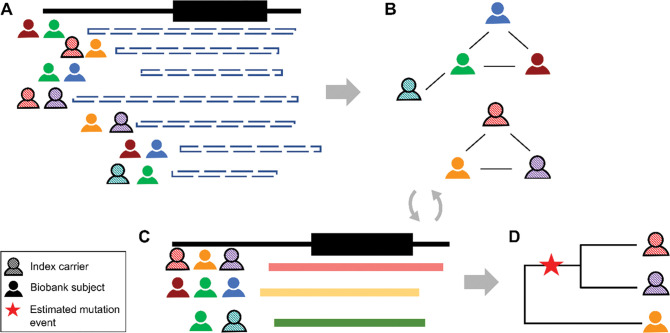
The DRIVE tool for local IBD clustering. This new tool identifies groups of people who share an IBD segment spanning a specific genomic region (in this study, the gene *KCNE1*). **A.** DRIVE first selects the pairwise IBD segments spanning the target gene/variant among clinic samples and biobank subjects. **B.** DRIVE uses a random walk approach to cluster subjects who share the same haplotype. **C.** DRIVE repeats the clustering steps for large and sparse clusters. **D.** The inverse of the IBD segment lengths is used to represent genetic distance in a phylogenetic dendrogram. Sequence data can be integrated with the dendrogram to infer where in the family history of the genomic region the mutation event occurred (red star).

The purpose of local IBD clustering is to identify sets of people who share an identical IBD segment spanning a specific genomic region (gene) or position (genetic variant). Since current IBD detection tools only report pairwise IBD sharing, we developed a new tool, DRIVE, to link individuals into connected graphs based on pairwise IBD sharing (Figure 1). DRIVE is implemented in python3.6 (https://github.com/belowlab/drive). To find potential *KCNE1* p.Asp76Asn BioVU carriers, we used DRIVE to identify all pairwise IBD segments greater than 3 cM in length spanning *KCNE1* (Figure 1A), and then conducted a three-step random walk approach, using segment length as the probability weight, to identify IBD clusters (Figure 1B). Random walk is an efficient approach for determining highly connected clusters within large and sparse networks, such as those derived from IBD sharing among biobank participants.^29^ Networks of close relatives would be expected to be highly connected, however spurious networks or networks connecting very short segments (due to distant relatedness) may be more sparsely connected. Therefore, for large (n > 30) and sparse (proportion of connected edges < 0.5) clusters, we ran additional random walks to split the clusters into smaller but more highly connected sub-clusters, with a maximum of five iterations of this process. We then carried each local IBD cluster containing clinic subjects forward in analysis of potential *KCNE1* p.Asp76Asn carrier clusters (Figure 1C). Finally, we used the inverse of shared IBD length to represent the local familial distance for each pair, and drew phylogenetic dendrograms with FastME 2.0 (Figure 1D).^30^

### Whole exome-sequencing validation

The presence of *KCNE1* p.Asp76Asn in each BioVU subject identified as sharing a p.Asp76Asn haplotype was assessed by exome sequencing on an Illumina NextSeq 500 with 150 bp paired-end reads following a standard Illumina protocol by the sequencing core at VANTAGE (Vanderbilt Technologies for Advanced Genomics). Sequencing quality control was conducted by fastp to filter out short and low-quality reads, following established approaches.^31^ Reads that passed QC were aligned to the hg38 human genome reference by BWA2,^32^ and the exome-wide variants were called by GATK following standard best practices.^33^

### Mutation age estimation

Including the newly identified BioVU p.Asp76Asn carriers, the clusters showed evidence of sharing a small haplotype at *KCNE1*. Because this suggests co-inheritance from a common ancestor, we randomly selected two from each cluster and estimated the age of the mutation event using the recombination clock model within the Genealogical Estimation of Variant Age (GEVA) tool.^34^ The length of shared segments spanning the target variant were used to estimate the time to the most recent common ancestor with the European ancestry 1000 Genomes data as reference.^20^ GEVA then estimates the age of the mutation event by comparing the estimated time to the most recent common ancestor among the pair of subjects that carry the target variant relative to pairs in which only one subject carries the target variant and the other one does not.

### Genome imputation and polygenic risk score calculation

Genome-wide genotype imputation was performed on p.Asp76Asn carriers, along with 3000 BioVU controls to stabilize the imputation process. The imputation controls were randomly selected from the subset of BioVU EA subjects with MEGA genotypes that passed QC, as described above. The subset of single nucleotide polymorphisms (SNPs) passing quality control that overlapped between p.Asp76Asn carriers and the imputation controls were used in imputation on the Michigan Imputation Server^35^ using the European ancestry Haplotype Reference Consortium version r1.1 2016 reference haplotypes.^36^ SNPs with low imputation quality (R^2^<0.3) were filtered before further analysis.

We then used the PRS for QTc developed by Nauffal et al.^37^ This PRS comprises 1,110,494 SNPs, and there were 1,110,297 overlapping SNPs between the PRS and our imputed genomes. Using the *score* function in PLINK, we calculated the PRS from the imputed genetic data of each carrier and control subject.

### Electronic health records review

ECGs obtained during routine clinical care were available for all clinic carriers, and for 13/22 biobank carriers. The QT interval corrected for heart rate (QTc) was calculated using the Bazett formula. For clinic and BioVU p.Asp76Asn carriers, QTc measurements were only used if the ECG was sinus rhythm or atrially paced, had a QRS duration <110 msec and a heart rate 50–100 bpm, and was not obtained while the subject was inpatient or prescribed QT-prolonging drugs (Table S1).

Arrhythmia diagnoses in each carrier’s deidentified EHR were determined using ICD-9 and ICD-10 codes (Table S2). Additionally, a text-search of each carrier’s EHR was performed for the terms “cardiac arrest,” “long QT,” “LQT,” “PMVT,” “polymorphic ventricular tachycardia,” “SCD,” “seizure,” “sudden cardiac death,” “syncope,” “Torsade(s),” “TdP,” “ventricular fibrillation,” “V fib,” and “VF;” any matches were manually reviewed by a physician to confirm the diagnosis.

ECG controls were defined as the BioVU subjects who (1) clustered with 1000 Genomes subjects from the European superpopulation (European Ancestries-like, EA) in genetic principal components analysis and passed genomic QC (described above) (n=69,819) and (2) had an ECG that met the following criteria: read as “normal,” in sinus rhythm, with QRS duration 65–110 msec and heart rate 50–100 bpm, obtained in an outpatient setting with no inpatient visits immediately before or after, with no QT-prolonging drugs prescribed at the time of the ECG (see Table S1 for list of drugs^38^), from a subject with no prior heart disease ICD-9 or ICD-10 codes and potassium within the VUMC lab normal limits. Controls were restricted to the EA subset because all clinic carriers self-described as “White” and clustered with 1000 Genomes subjects from the European super-population, as above. One BioVU p.Asp76Asn carrier overlapped with this set, and was removed from the control group. This resulted in an ECG control group of 3,435 genotyped individuals (2218 females,1217 males) with an ECG meeting our criteria.

### Statistical analysis

Statistical tests were performed using R version 4.2.1^39^ Regression modeling used the *rms* package in R.^40^ For continuous variables, parametric testing was used if each group had >10 members and satisfied the Shapiro-Wilk test; otherwise, non-parametric testing was used. All parametric tests were two-tailed. *P-value<*0.05 was considered statistically significant. For categorical variables, the Fisher’s exact test was used due to small sample sizes.

## Results

### Distant relatedness in a local cluster of p.Asp76Asn probands

Nine p.Asp76Asn probands were referred to the Genetic Arrhythmia Clinic for LQTS evaluation and treatment. Cascade screening identified three additional p.Asp76Asn carriers among the probands’ first-degree relatives, resulting in 12 clinic subjects confirmed to carry p.Asp76Asn (Table 1). None of the probands were known to be related. All probands had experienced syncope, 5 had a QTc >480 ms absent QT-prolonging drugs or electrolyte abnormalities, 5 had an implanted cardioverter-defibrillator (ICD), and 3 had documented Torsades de Pointes (TdP), the polymorphic ventricular tachycardia seen in LQTS. Among the 3 carrier family members, 1 had a QTc >480 ms, 1 had a primary prevention ICD, none had a history of syncope, and none had experienced TdP.

**Figure 2.**
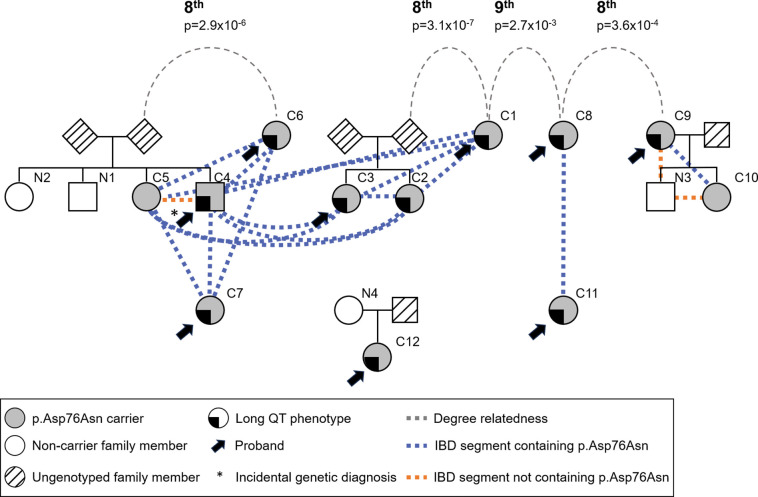
p.Asp76Asn probands are distantly related. Analysis of genome-wide IBD sharing in the clinic p.Asp76Asn carriers was used to reconstruct all known pedigrees of first-degree relatives and to identify previously unknown eighth to ninth degree relatedness among these pedigrees and three of the probands without close relatives. It is possible that more distant relatedness exists between the families that is beyond the limit of detection of existing tools (~9^th^ degree). The colored, dashed lines indicate shared IBD segments (≥ 3 cM) spanning *KCNE1*, both those harboring p.Asp76Asn (blue) and not (orange). The “long QT phenotype” was defined simply as having QTc > 480 in the absence of QT-prolonging drugs or electrolyte derangements, a documented history of Torsades de Pointes, or recurrent, unexplained syncope. IBD = identical by descent

We estimated pairwise relatedness by the global proportion of IBD sharing and the distribution of shared IBD segments, using PRIMUS and ERSA software as described, enabling reconstruction of the three known nuclear pedigrees. Using PADRE, we identified previously unknown eighth to ninth degree relatedness among these pedigrees and three of the probands without close relatives (Figure 2). For reference, third cousins are eighth degree relatives. This relatedness supports the hypothesis that a local founder event underlies the comparatively high p.Asp76Asn frequency in Tennessee.

### Using relatedness to identify p.Asp76Asn carriers in BioVU

**Figure 3.**
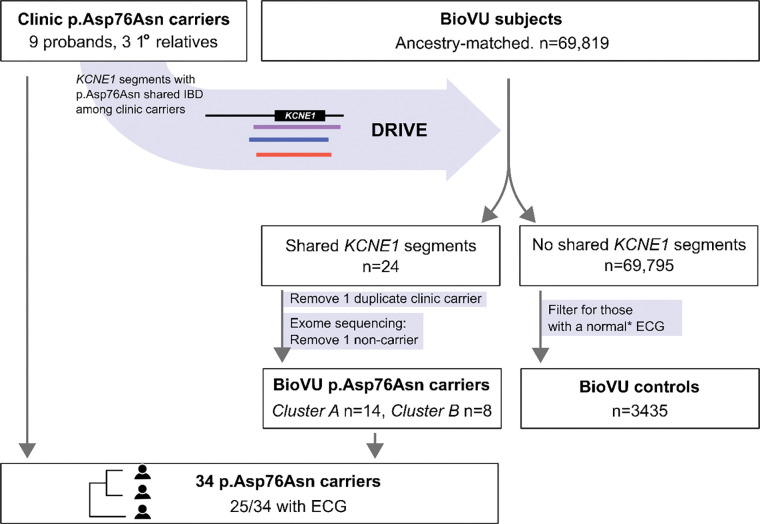
Study design. Clinic p.Asp76Asn carriers comprise nine probands seen at the VUMC Genetic Arrhythmia Clinic and three related carriers identified through cascade screening. IBD-based genotype inference using the DRIVE tool was deployed in VUMC BioVU, which links the deidentified electronic health record to genomic data, to identify individuals who shared chromosomal segments at *KCNE1* with the clinic carriers*.* Exome sequencing confirmed p.Asp76Asn carrier status in 22/23 biobank individuals identified via IBD, resulting in total of 34 p.Asp76Asn carriers at a single center. BioVU subjects without shared *KCNE1* segments who also had at least one ECG that met inclusion criteria detailed in Methods (*) were used as the control group. IBD = identical by descent, DRIVE = Distant relatedness for Identification and Variant Evaluation, ECG = electrocardiogram.

**Figure 4.**
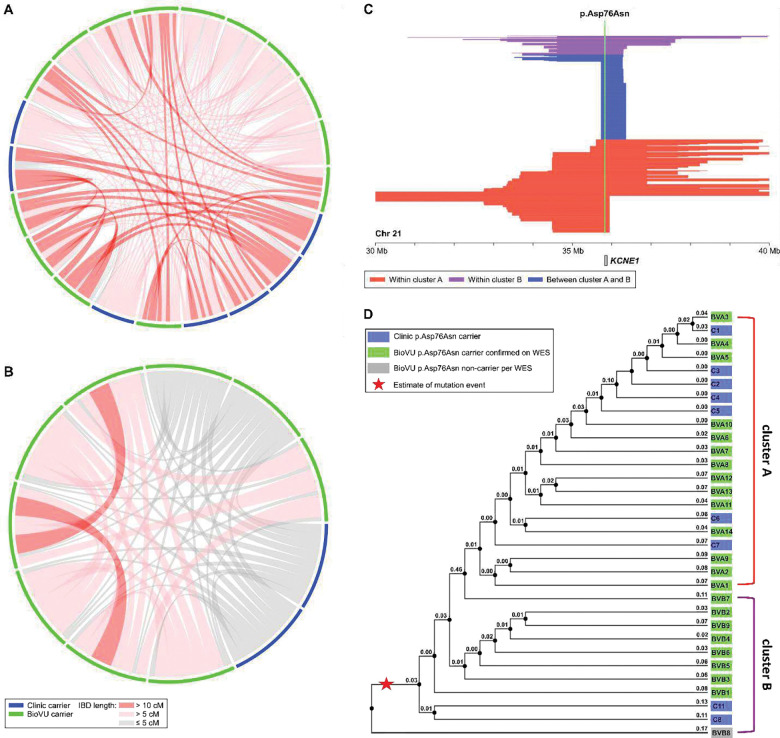
IBD clustering revealed distantly related subjects in the biobank. Local IBD sharing at *KCNE1* was analyzed between the 12 clinic carriers and the 69,819 subjects in BioVU of European descent. This identified two clusters containing known carriers, with an additional 23 subjects sharing the same chromosomal segment at *KCNE1.* Of the 23 BioVU subjects, exome sequencing confirmed p.Asp76Asn in 22. In the connection plots for cluster A (**A**) and cluster B (**B**), individuals are represented by the segments along the periphery. Shared chromosomal segments at *KCNE1* between individuals are indicated by the red and gray connectors. **C)** Illustration of the length and position of the shared segments spanning *KCNE1* among members of cluster A (red), members of cluster B (purple), and in members of both cluster A and B (blue). **D)** The dendrograms for cluster A and cluster B represent generational distance between subjects based on the inverse of the length of the IBD segments underlying *KCNE1* (labeled). All clinic subjects (“C” prefix, blue) had p.Asp76Asn carriership determined by clinical-grade commercial testing.BioVU subjects (“BV” prefix) in green had p.Asp76Asn carriership confirmed by whole exome sequencing (WES), and the BioVU subject in gray was found not ot carry p.Asp76Asn on WES. The red star indicates the possible mutation event on the shared haplotype. The annotated branch length in the dendrograms for cluster A and cluster B represents the local familial distance between subjects, estimated as the inverse of the length of the IBD segments underlying *KCNE1* (numeric label on each node). IBD = identical by descent.

We analyzed the 12 clinic carriers in conjunction with the BioVU EA genotyped population (Figure 3). In this merged dataset, 582,671 long IBD segments (>3 cM) were detected spanning *KCNE1*. Using DRIVE, we identified 12,356 IBD clusters at the *KCNE1* locus with at least three members, including two clusters containing at least two confirmed carriers from the clinic samples. The first cluster (cluster A) included 7 clinic carriers and 14 BioVU subjects, and the second cluster (cluster B) included another 2 clinic carriers and 9 BioVU subjects. In cluster A, 82.9% of pairs shared IBD segments >3 cM (Figure 4A) spanning *KCNE1*, with an average segment length of 12.3 cM. In cluster B, 88.9% of pairs shared IBD segments spanning *KCNE1*, with an average length of 6.0 cM (Figure 4B). To explore more distant relatedness, we analyzed short IBD segments (>1 cM) shared across the clusters. In total, we identified 165 short IBD segments between members of cluster A and B (Figure 4C), with an average length 1.41 cM, suggesting the two clusters are distantly related, and motivating joint analysis of variant age (Figure 4D). Whole exome-sequencing confirmed p.Asp76Asn heterozygosity in 22 of the 23 BioVU subjects in the clusters. Our expanded dataset included 34 mutation carriers, 12 from clinic and 22 from BioVU. No other BioVU subjects were found to share the IBD segment (>3 cM) harboring the p.Asp76Asn variant. We applied a recombination clock approach, GEVA, to estimate the age of p.Asp76Asn. Using two BioVU carriers from each cluster and unrelated Europeans from 1000 Genomes, GEVA estimated that the mutation occurred 46 generations ago.

### Cardiac events in p.Asp76Asn carriers

We summarized the clinical characteristics of the 34 carriers in Table 1. EHR review identified four with documented TdP arrhythmia. Three were subsequently seen in the Genetic Arrhythmia clinic and are members of the clinic cohort. Of these three, one suffered an unprovoked cardiac arrest and was successfully resuscitated. The second suffered an in-hospital TdP arrest following albuterol administration. The third had self-limited TdP observed on telemetry, without arrest. TdP was documented in one BioVU carrier, attributed to use of sotalol, a QT-prolonging drug. Five clinic carriers had a documented first-degree relative with SCD or Torsades arrest. EHR review identified syncope, which is non-specific but indicates high risk in patients with LQTS,^41,42^ in 15/34 carriers. Compared to the clinic carriers, a smaller proportion of BioVU carriers were female, and fewer BioVU carriers had a history of syncope, had a first-degree relative with SCD or Torsades arrest, were on beta blockade, or had an ICD. BioVU carriers had a shorter presenting QTc compared to clinic carriers (432 msec vs. 463 msec, respectively, *p-value*=0.019).

### The QTc is prolonged in p.Asp76Asn carriers

**Figure 5.**
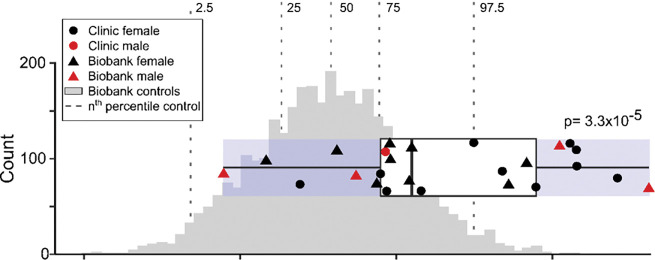
The QTc in p.Asp76Asn carriers compared to population controls. ECGs meeting criteria detailed in Methods were available for all clinic carriers, and for 13 of 22 biobank (BioVU) carriers. The ancestry-matched BioVU controls with genotype and ECG data (n=3,435) were selected as detailed in Methods. For both carriers and controls, if multiple ECGs were available for a subject, the maximum QTc meeting inclusion criteria was used. Male QTcs were adjusted to female sex by adding 11.3 msec (derived from the difference between males and females in the control group when adjusted for age and PRS). The QTc was prolonged in carriers (465±6.2 msec, n=25) compared to controls (429±23.3 msec, n= 3,435; *p-value*=3.3×10^−5^). The boxplot shows the three quartiles (25%, 50%, and 75%) of the carriers. *P-*values indicate the result of the Welch unequal variances t-test between carriers and controls, with *p-value*<0.05 considered significant.

Among the 25 p.Asp76Asn carriers with an ECG, representing both clinic and BioVU subjects, 40% of female carriers (8/20) had a QTc >480 msec, compared to 1.5% of female controls. In male carriers, 40% (2/5) had a QTc >480 msec, compared to 0.7% of male controls. The QTc, adjusted for sex, was longer in carriers (465±36 msec) compared to population controls (429±23 msec, *p-value*=3.3×10^−5^) (Figure 5). To assess the effect of p.Asp76Asn in a non-referral population, we then restricted the analysis to the subset of carriers from BioVU. In this BioVU-only group, the QTc remained longer than that of population controls (456±38 msec vs. 429±23 msec, *p-value*=0.023).

### Polygenic risk in p.Asp76Asn carriers

**Figure 6.**
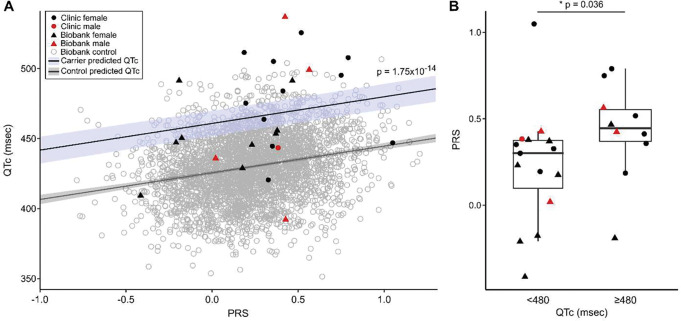
Polygenic risk for QT prolongation and p.Asp76Asn carrier status contribute additively to QT prolongation. **A)** The PRS for the QTc was calculated for each carrier (clinic n=12, BioVU n=13) and for the BioVU controls (n=3,435). A multivariable linear regression analysis for the QTc as a function of p.Asp76Asn carrier status, PRS, age, and sex (Table S3) predicted that p.Asp76Asn carriers have an average 35.3 msec longer QTc compared to controls (*p-value*=1.75×10^−14^). The predicted QTc as a function of PRS is shown for carriers (blue line) and non-carriers (gray line). **B)** Carriers with a prolonged QTc have a higher PRS than carriers with a normal QTc. The maximum QTc meeting inclusion criteria was used, and was adjusted for age and sex. The *p*-values indicates the result of Mann Whitney U test comparing carriers with maximum QTc <480, and ≥480 msec. *P-value* <0.05 was considered significant. ECG = electrocardiogram, PRS = polygenic risk score.

We assessed whether the QTc interval PRS contributed to QTc variability in p.Asp76Asn carriers and in controls (Figure 6A). A multivariable linear regression analysis for the QTc as a function of p.Asp76Asn carrier status, PRS, age, and sex was performed (Table S3). These variables were all statistically significant in this model, which showed p.Asp76Asn carriers have an average 35.3 msec longer QTc compared to controls (*p-value*=1.75×10^−14^). This supports the conclusion that p.Asp76Asn carriers have longer QTc intervals than controls, even when polygenic risk for QT prolongation is considered. We additionally tested the interaction between PRS and p.Asp76Asn on QTc (Table S3), and did not observe a significant effect beyond additive interaction (*p-value*=0.194).

We then assessed the relationship between the QTc PRS and QTc among p.Asp76Asn carriers. QTc intervals were adjusted for age and sex in this analysis; QTc is adjusted to female sex and age 47 (mean age of the clinic subjects), based on the regression model derived from the control population (Table S3). The QTc PRS was different between p.Asp76Asn carriers with a prolonged (≥480 msec) QTc versus those without (QTc<480 msec), with a median PRS of 0.45 (interquartile range (IQR) 0.37–0.55) compared to 0.30 (IQR 0.099–0.37), respectively (*p-value*=0.036, Figure 6B). We found a trend towards higher PRS in individuals with a history of Torsades (*p-value*=0.073), and in clinic carriers compared to BioVU carriers (*p-value*=0.053) (Figure S2A,B). The diagnosis of syncope was not associated with PRS (Figure S2C).

## Discussion

Here we leverage patterns of sharing within the genome to make three key findings. First, we characterize both known and unknown relatedness within clinic patients identified as p.Asp76Asn carriers after a cardiac event (in them or a close relative) prompted referral to the Genetic Arrhythmia Clinic. The observed relatedness among carriers indicates that many carriers are descended from a common ancestor, suggesting a local founder event explains the excess of carriers of the rare p.Asp76Asn variant. Second, we present a novel approach in a large biobank that enabled discovery of additional p.Asp76Asn carriers who may be at risk of developing or have a missed diagnosis of LQT5. Third, we leverage these additional carriers from a non-referral population to evaluate the penetrance of LQT5 in p.Asp76Asn carriers and assess the role of QTc genetic risk factors in combination with p.Asp76Asn .

Both common and rare variants contribute to genetic risk in cardiovascular disease. Although the monogenic or Mendelian diseases are individually rare, in aggregate they impact 7–8% of the United States population.^43^ Since these diseases may cause high morbidity and mortality rates, early diagnosis, especially when impactful interventions are available, is especially important. Genomic sharing due to relatedness provides a powerful but underutilized approach to detect ungenotyped or poorly genotyped variation and estimate its effect. For example, Belbin *et al.*^7^ used IBD to identify multiple shared chromosomal segments associated with short stature in Puerto Ricans in an ethnically-diverse biobank, and follow-up analyses demonstrated that homozygous carriage of a rare variant in the collagen gene *COL27A1* was responsible. Although patterns of genomic sharing have been used in disease gene discovery before, the approach is not often implemented and is limited by the lack of effective tools for biobank-scale analysis.

Ameliorating this challenge, we developed a new approach, DRIVE, to perform biobank-scale analysis and discover 22 p.Asp76Asn carriers. This allowed us to determine that p.Asp76Asn significantly prolongs the QT interval, even in a non-referral population. In assessing pathogenicity, a commonly used criterion is allele frequency in large public datasets; however, the lack of associated phenotypic data limits the informativeness of these analyses.^44^ In the Genome Aggregation Database,^24^ the frequency of *KCNE1* p.Asp76Asn is very rare with a minor allele frequency of 0.00011 in Europeans, and 0.000067 overall. Thus, as with other rare variants, determination of pathogenicity and penetrance of p.Asp76Asn requires additional evaluation such as functional data and association with disease-related human phenotypes. In functional studies, p.Asp76Asn exerts profound dominant negative effects on I_Ks._^9^ Our human phenotypic data add further strength to the conclusion of the International Consortium that *KCNE1* variants can be monogenic causes of long QT syndrome, but penetrance is incomplete.^17^

Finally, we explored factors that modify penetrance of p.Asp76Asn. Polygenic variation has been shown to modulate penetrance in tier 1 monogenetic conditions^45^ as well as a rare, monogenic *SCN5A* arrhythmia syndrome.^46^ Similarly, we find an additive interaction between p.Asp76Asn and QT variation across the genome, as measured by a QT PRS, that suggests the risk effect of p.Asp76Asn is significantly modified by the rest of the genome. This work both illustrates a new paradigm for studying the effects of ungenotyped or poorly genotyped variation among cryptic relatives in regionally sampled datasets, DRIVE, and demonstrates the power of rare variant detection and evaluation in non-referral populations to improve estimates of variant effects.

In summary, our method enables identification of rare variant carriers in non-referral populations. Panel or whole genome sequencing in affected probands enables comprehensive identification of carriers of pathogenic or likely pathogenic variants in Mendelian disease genes; however, failure to detect undiagnosed carriers of rare, pathogenic variants results in missed opportunities for preventative care, and undertreatment of disease. Furthermore, failure to detect carriers in general populations with dense associated phenotypic information limits the study of rare, inherited diseases due to low sample sizes and ascertainment bias, which prevents accurate estimates of pathogenicity, penetrance, and relevant modifying effects. Most publicly available biobank data is array-based or exome-sequenced, rendering much of the functional variation unassessed. Thus, discovering subjects with shared chromosomal segments at a Mendelian disease locus known to harbor a disease-causing mutation in at least one individual holds the promise of identifying other carriers, even when their sequencing data is not available.

### Limitations

Although this study represents the largest analysis to date of the role of p.Asp76Asn in general populations, there are limitations to using the EHR to evaluate its effects. While none of the BioVU p.Asp76Asn carriers have a LQT5 diagnosis in their record, a diagnosis may have been made outside Vanderbilt. Similarly, no BioVU carriers had documentation of a first-degree family member with SCD; this could be due to insufficient family history documentation in the EHR, in contrast to the clinic carriers seen in a Genetic Arrhythmia clinic, where obtaining family history is a priority. Further, our estimation of the effects of QTc polygenic risk in carriers was limited to those with an ECG, which reduced our power and may introduce ascertainment bias. Finally, confounding by population haplotypes, genotyping error, and genotype data density limits the ability to accurately estimate the degree of relatedness using very short segments, and as a result our ability to estimate segments smaller than 3 cM, or relationships more distant than 8^th^ or 9^th^ degree.

### Conclusion

We introduce a new approach to leveraging distant relatedness to identify rare variant carriers and use this approach to identify p.Asp76Asn carriers who are undiagnosed for or at risk of developing LQT5. We use the set of non-referral carriers to improve estimation of p.Asp76Asn penetrance, detect polygenic effects modifying pathogenesis, and estimate mutation age. This demonstrates that analysis of shared chromosomal segments in large numbers of subjects with dense phenotypic data enables the discovery of mutation carriers and evaluation of disease loci.

## Figures and Tables

**Table 1.** Demographic and clinical features of p.Asp76Asn carriers

	All carriers (n=34)	Clinic (n=12)	BioVU (n=22)	*P*-value

**Demographics**				
Age at first ECG (year)	44.2 ± 19.7	42.5 ± 20.1	45.7 ± 19.9	0.69
Female, n (%)	21 (62%)	11 (91.7%)	10 (45.5%)	0.011
**Phenotype**				
QTc on first ECG (msec)	447 ± 32.9	463 ± 36.1	432 ± 21.7	0.019
Max QTc (msec)	463 ± 36.7	475 ± 33.6	453 ± 37.4	0.13
Syncope, n (%)	15 (44%)	9 (75%)	6 (27.2%)	0.012
Torsade or CA, n (%)	4 (12%)	3 (25%)	1 (4.55%)	0.12
1° relative with SCD or Torsade arrest	5 (15%)	5 (41.7%)	0 (0%)	0.0028
**Treatment**				
Beta blockade, n (%)	12 (35.3%)	9 (75%)	3 (13.6%)	1.7 ×10^−4^
ICD, n (%)	6 (17.6%)	6 (50%)	0 (0%)	6.9 ×10^−4^

Data are presented as mean ± standard deviation or n (%). *P*-values compare clinic carriers to BioVU carriers, using the Fisher’s exact test for categorical variables and the Mann Whitney U test for continuous variables. For age at first ECG, if no ECG was available, the current age was used. CA = cardiac arrest, SCD = sudden cardiac death, ICD= implantable cardioverter- defibrillator.

## Data Availability

DRIVE is available at https://github.com/belowlab/drive. The clinical and genetic data supporting the current study have not been deposited in a public repository because of the risk of subject re-identification in this small sample size.
